# Cytological Evaluation of Oral Epithelial Changes in Apparently Healthy Oral Mucosa Among Smokers and Spicy Food Consumers

**DOI:** 10.7759/cureus.106786

**Published:** 2026-04-10

**Authors:** Faris M Elmahdi, Shadia Abdulaziz, Amal Ayed, Dunyh Safar, Wejdan Mohammed, Lina A Salim, Sara Altom

**Affiliations:** 1 Basic Sciences, Al-Rayan National College of Medicine, Madinah, SAU; 2 Medicine and Surgery, Al-Rayan National College of Medicine, Madinah, SAU; 3 Anesthesiology, Al-Rayan National College of Medicine, Madinah, SAU

**Keywords:** cytological abnormalities, e-cigarette, oral mucosa, papanicolaou stain, saudi arabia, shisha, spicy food

## Abstract

Background

Tobacco use remains a leading cause of morbidity worldwide, with increasing use of alternative forms such as shisha and e-cigarettes. Concurrently, dietary habits such as frequent spicy food consumption are common in many cultures. While the systemic effects of smoking are well documented, the localized cytological impact of both smoking and repeated dietary irritation from spicy foods on apparently healthy oral tissues requires further comparative investigation. Hence, this study aimed to evaluate and compare cytological alterations in the oral mucosa among users of cigarettes, shisha, and e-cigarettes, as well as frequent spicy food consumers, in comparison with a healthy control group.

Methodology

A cross-sectional study was conducted among 400 Saudi adults (300 smokers, 50 frequent spicy food consumers, and 50 healthy controls) between January and May 2025. Smoking exposure was further characterized by duration, frequency, and pack-years. Buccal mucosal smears were collected from standardized sites and stained using the Papanicolaou technique. Cytological abnormalities, including inflammation, microbial infection, cellular atypia, and binucleation, were assessed using predefined criteria. Statistical analysis was performed using SPSS version 22 (IBM Corp., Armonk, NY, USA), with significance set at p-values <0.05.

Results

Cytological abnormalities were significantly more frequent among smokers (48%) and spicy food consumers (34%) compared with controls (20%) (p < 0.05). Among smoking subgroups, e-cigarette users demonstrated a higher prevalence of nuclear alterations, including cellular atypia (15%) and binucleation (8.8%); however, this finding should be interpreted with caution given the smaller subgroup size (n = 80). In contrast, inflammatory changes were most commonly observed among spicy food consumers (26%), followed by cigarette smokers (23.3%).

Conclusions

Both smoking and frequent spicy food consumption are associated with cytological alterations in the oral mucosa. Smoking, including e-cigarette use, appears to be associated with nuclear alterations, while spicy food consumption is primarily associated with inflammatory changes. These findings should be interpreted in light of the cross-sectional design and potential confounding factors. Further longitudinal studies are required to clarify the clinical significance of these observations.

## Introduction

Tobacco use remains a major contributor to preventable morbidity and mortality worldwide. According to the World Health Organization, tobacco consumption is responsible for more than 8 million deaths annually, including both direct smokers and individuals exposed to secondhand smoke [1]. Beyond its systemic health consequences, such as cardiovascular diseases, chronic respiratory disorders, and multiple forms of cancer, tobacco smoking also exerts significant local effects on the oral cavity. Because the oral mucosa is the primary site of contact with tobacco smoke, it is particularly vulnerable to the thermal, chemical, and mechanical insults produced during smoking [2].

Numerous studies have documented the detrimental effects of smoking on oral health. Common oral manifestations associated with tobacco use include gingival inflammation, periodontal disorders, impaired wound healing, accumulation of dental plaque, oral malodor, and discoloration of both oral tissues and teeth [3]. More importantly, tobacco smoking is recognized as a significant risk factor for oral potentially malignant disorders as well as oral squamous cell carcinoma. Lesions such as leukoplakia and erythroplakia are strongly associated with chronic tobacco exposure and may progress to malignancy if early cellular alterations are not detected [4,5]. Cigarette smoke contains a complex mixture of more than 7,000 chemical substances, including at least 70 known carcinogens such as nitrosamines, formaldehyde, and polycyclic aromatic hydrocarbons. These compounds can induce DNA damage and promote mutagenic changes within oral epithelial cells, thereby increasing the risk of malignant transformation [6].

In addition to tobacco use, various dietary habits may influence the health of the oral mucosa. Frequent consumption of spicy foods is common in many cultures and has been suggested to act as a source of repeated mucosal irritation. Spicy foods often contain biologically active compounds such as capsaicin, which can activate transient receptor potential vanilloid 1 (TRPV1) receptors on sensory nerve endings and induce neurogenic inflammatory responses within oral tissues [7]. Prolonged or repeated exposure to such irritants may lead to epithelial adaptation, increased keratinization, or other cytological alterations within the oral mucosa [8]. Although these effects are generally considered less harmful than tobacco exposure, persistent irritation may still contribute to subtle cellular changes that could be detectable through cytological examination.

Importantly, the inclusion of frequent spicy food consumption in this study was not intended to equate its risk magnitude with tobacco exposure, but rather to represent a commonly encountered non-tobacco source of repeated mucosal irritation. Comparing these exposures allows for a broader understanding of how different types of chronic irritants may influence oral epithelial cytology.

Oral exfoliative cytology has emerged as a valuable, non-invasive technique for evaluating epithelial cell morphology and identifying early cellular abnormalities. This method allows the detection of inflammatory changes, nuclear alterations, microbial infections, and early dysplastic features before clinically visible lesions develop [9]. Because of its simplicity and cost-effectiveness, exfoliative cytology has become an important screening tool for assessing oral mucosal health, particularly in populations exposed to potential risk factors such as tobacco and dietary irritants.

Despite extensive research on the effects of smoking on oral tissues, limited comparative studies have examined the differential impact of various forms of smoking (cigarette, shisha, and e-cigarette) alongside non-tobacco irritants such as frequent spicy food consumption on oral epithelial cells. Understanding the cytological changes associated with these lifestyle habits may help identify early mucosal alterations that occur even in clinically normal oral tissues.

Therefore, this study aimed to evaluate and compare cytological alterations in the apparently healthy oral mucosa among individuals exposed to different risk factors, including cigarette smoking, shisha use, e-cigarette use, and frequent consumption of spicy foods, in comparison with individuals without these habits. The study also aimed to assess the potential role of oral exfoliative cytology in detecting early epithelial changes.

## Materials and methods

Study design and participants

This cross-sectional study was conducted between January and June 2025 in Madinah, Saudi Arabia. A total of 400 apparently healthy volunteers were enrolled and categorized into the following three main groups: 300 smokers, 50 frequent spicy food consumers, and 50 healthy controls. The smoker group was further subdivided into cigarette smokers (n = 120), shisha users (n = 100), and e-cigarette users (n = 80).

Sample size was calculated using Epi Info software (Version 7.2; Centers for Disease Control and Prevention, Atlanta, GA, USA), based on a 95% confidence level and a 5% margin of error.

Participants included Saudi adults aged 18 to 85 years who were in good general health. The spicy food group consisted of non-smokers who reported consuming spicy food at least five times per week, reflecting repeated exposure to capsaicin-containing foods. The control group included non-smokers who did not consume spicy foods regularly. Individuals with pre-existing oral lesions, systemic diseases (e.g., diabetes mellitus), non-Saudi participants, and those under 18 years of age were excluded.

Assessment of smoking exposure

Detailed smoking exposure data were collected using a structured questionnaire, including age at smoking initiation, duration of smoking (years), number of cigarettes per day, and cumulative smoking exposure expressed as pack-years. These measures were used to better characterize smoking intensity and long-term exposure among participants.

Sample collection

Buccal smear samples were obtained from the oral mucosa for cytological assessment. Using a sterile wooden tongue depressor, exfoliated cells were collected from the dorsal surface of the tongue and the bilateral buccal mucosa. The collected material was evenly spread onto clean glass slides and immediately fixed in 95% ethyl alcohol. Prepared smears were transported to the histopathology laboratory at Al-Rayan College of Medicine, Saudi Arabia, for further processing and analysis.

Papanicolaou staining

Following ethanol fixation, the smears were hydrated through a descending series of ethanol concentrations (95% to 70%), each for two minutes. Nuclear staining was performed using Harris hematoxylin for five minutes, followed by rinsing in distilled water. Differentiation was performed using 0.5% aqueous hydrochloric acid for 10 seconds, followed by rinsing and bluing in alkaline water.

The smears were then dehydrated through an ascending series of ethanol concentrations (70% to 95%), each for two minutes. Cytoplasmic staining was performed using Papanicolaou Orange G6 for two minutes, followed by rinsing in 95% ethanol, and subsequent staining with EA-50 solution for three minutes. After dehydration in absolute ethanol, the smears were cleared in xylene and mounted using dibutylphthalate polystyrene xylene (DPX) [9].

Cytological evaluation

Pap-stained smears were examined under light microscopy for cytopathological abnormalities. The evaluated parameters included keratinization, cellular atypia, inflammatory changes, and microbial infection. Cytological alterations were identified based on predefined and standardized morphological criteria, including irregular cellular morphology, nuclear enlargement, increased nuclear-to-cytoplasmic ratio, and the presence of bi- or multinucleation. All slides were systematically examined under uniform magnification to ensure consistency in evaluation.

All cytological evaluations were performed by a single experienced observer trained in cytopathological assessment. The evaluation was conducted under standardized conditions using uniform diagnostic criteria to ensure consistency and minimize observer-related variability. The observer was blinded to participants’ exposure status during slide evaluation to reduce potential assessment bias. Although inter- and intra-observer reliability were not formally assessed, adherence to standardized criteria was maintained to enhance consistency and reduce observer-related bias.

Statistical analysis

Statistical analysis was conducted using SPSS Statistics for Windows, Version 22 (IBM Corp., Armonk, NY, USA). Categorical variables were presented as frequencies and percentages. A chi-square test of independence was used to assess the association between exposure groups and the presence of cytological abnormalities. Additional chi-square analyses were performed separately for each type of cytological abnormality (inflammation, cellular atypia, binucleation, and hyperkeratosis) across exposure groups to provide a more detailed evaluation. All statistical tests were two-tailed, and a p-value <0.05 was considered statistically significant. The assumptions of the chi-square test were verified, including adequate expected cell counts. Given the exploratory nature of the study, no formal adjustment for multiple comparisons was applied; therefore, the results were interpreted with caution.

Ethical considerations

Written informed consent was obtained from all participants before sample collection. The study protocol was reviewed and approved by the Research Ethics Committee of Al-Rayan Medical Colleges (AMC) (approval number: HA-03-M-122-090).

## Results

A total of 400 participants were included in this study. The majority of smokers and spicy food consumers were within the younger age groups (18-45 years), indicating a higher prevalence of these habits among younger adults. The highest proportion of smokers was observed in the 18-30-year age group. In contrast, participants in the control group were more evenly distributed across all age categories, including older age groups, as shown in Table 1.

**Table 1:** Age distribution of study participants across exposure groups.

Age group (years)	Smokers (n = 300)	Spicy food consumers (n = 50)	Control group (n = 50)
18–30	90 (30%)	20 (40%)	10 (20%)
31–45	110 (36.7%)	18 (36%)	12 (24%)
46–60	60 (20%)	8 (16%)	14 (28%)
>60	40 (13.3%)	4 (8%)	14 (28%)

Detailed smoking exposure characteristics are presented in Table 2, demonstrating variability in smoking initiation age, duration, intensity, and cumulative exposure.

**Table 2:** Smoking exposure characteristics among participants. Data are presented as mean ± standard deviation (SD), range, and median (interquartile range, IQR).

Parameter	Mean ± SD	Range	Median (IQR)
Age at smoking initiation (years)	19.3 ± 4.6	12–32	18.0 (16.0–22.0)
Duration of smoking (years)	19.2 ± 7.8	5–38	19.0 (13.0–25.0)
Cigarettes per day	22.4 ± 9.6	10–50	20.0 (15.0–28.0)
Pack-years	21.6 ± 13.4	2.5–75.0	19.0 (11.3–28.8)

**Figure 1:**
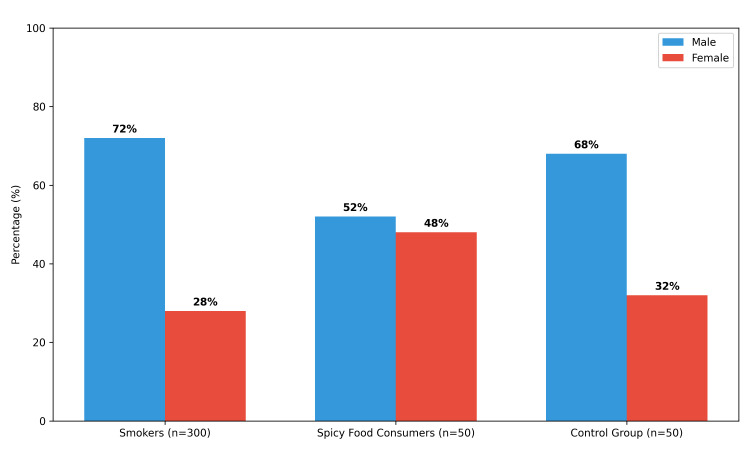
Gender distribution across study groups.

Gender analysis revealed a predominance of males among smokers, accounting for 216 (72%) of the group. In contrast, the spicy food consumer group demonstrated a more balanced gender distribution, with 26 (52%) males and 24 (48%) females. The control group showed a similar pattern, with males slightly more represented (34 (68%) vs. 16 (32%)). No statistically significant difference in gender distribution was observed between groups (χ² = 1.31, P = 0.27), as shown in Figure 1.

The prevalence of cytological abnormalities was highest among smokers (144/300; 48%), followed by spicy food consumers (17/50; 34%), while the control group demonstrated the lowest prevalence (10/50; 20%). This difference was statistically significant (χ² = 6.72, P = 0.018), as presented in Table 3.

**Table 3:** Comparison of cytological findings across study groups. Data are presented as n (%). Chi-square test was used. P-values <0.05 were considered statistically significant.

Group	Normal cells, n (%)	Abnormal cells, n (%)	χ²	P-value
Smokers (n = 300)	156 (52%)	144 (48%)	6.72	0.018
Spicy food consumers (n = 50)	33 (66%)	17 (34%)		
Control group (n = 50)	40 (80%)	10 (20%)		

Further analysis of cytological abnormalities revealed distinct patterns across exposure groups. E-cigarette users demonstrated a higher prevalence of nuclear alterations, including cellular atypia (12 (15%)) and binucleation (7 (8.8%)), compared with other smoking subgroups. In contrast, inflammatory changes were most frequently observed among spicy food consumers (13 (26%)), followed by cigarette smokers (28 (23.3%)) and shisha users (22 (22%)). Hyperkeratinization was observed across all groups with relatively comparable frequencies. These differences in the distribution of cytological abnormalities among exposure groups were statistically significant (overall χ² = 8.14, P = 0.032), as shown in Table 4.

**Table 4:** Distribution of cytological abnormalities by exposure group. Data are presented as n (%). The chi-square test was used. P-values <0.05 were considered statistically significant.

Abnormality type	Cigarette (n = 120)	Shisha (n = 100)	E-cigarette (n = 80)	Spicy food (n = 50)	χ²	P-value
Inflammation	28 (23.3%)	22 (22%)	15 (18.8%)	13 (26%)	8.14	0.032
Cellular atypia	8 (6.7%)	9 (9%)	12 (15%)	2 (4%)		
Binucleation	6 (5%)	5 (5%)	7 (8.8%)	3 (6%)		
Hyperkeratosis	10 (8.3%)	12 (12%)	10 (12.5%)	6 (12%)		

**Figure 2:**
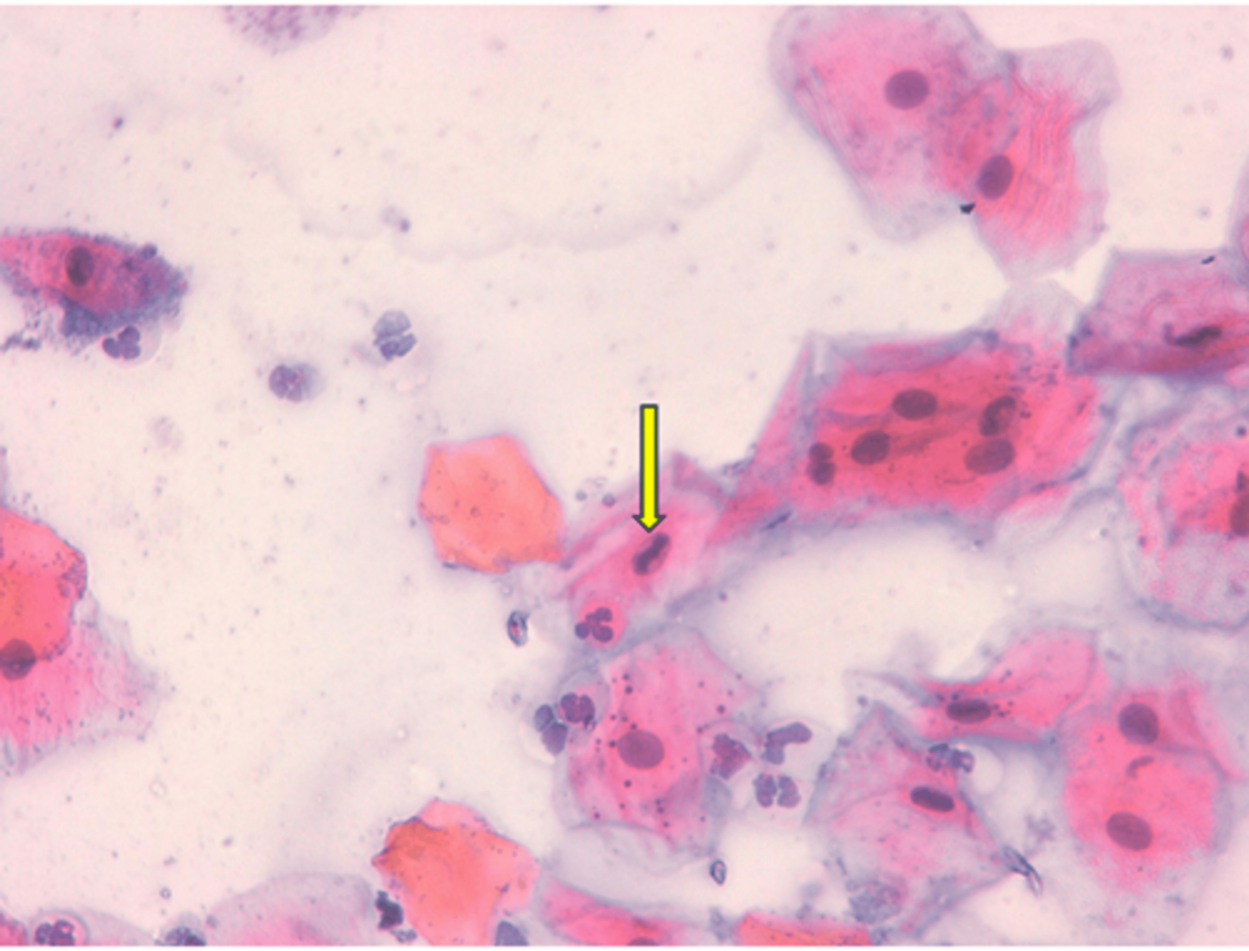
Microphotograph of a buccal mucosal smear stained using the Papanicolaou technique (×40), showing cellular atypia in shisha smokers.

**Figure 3:**
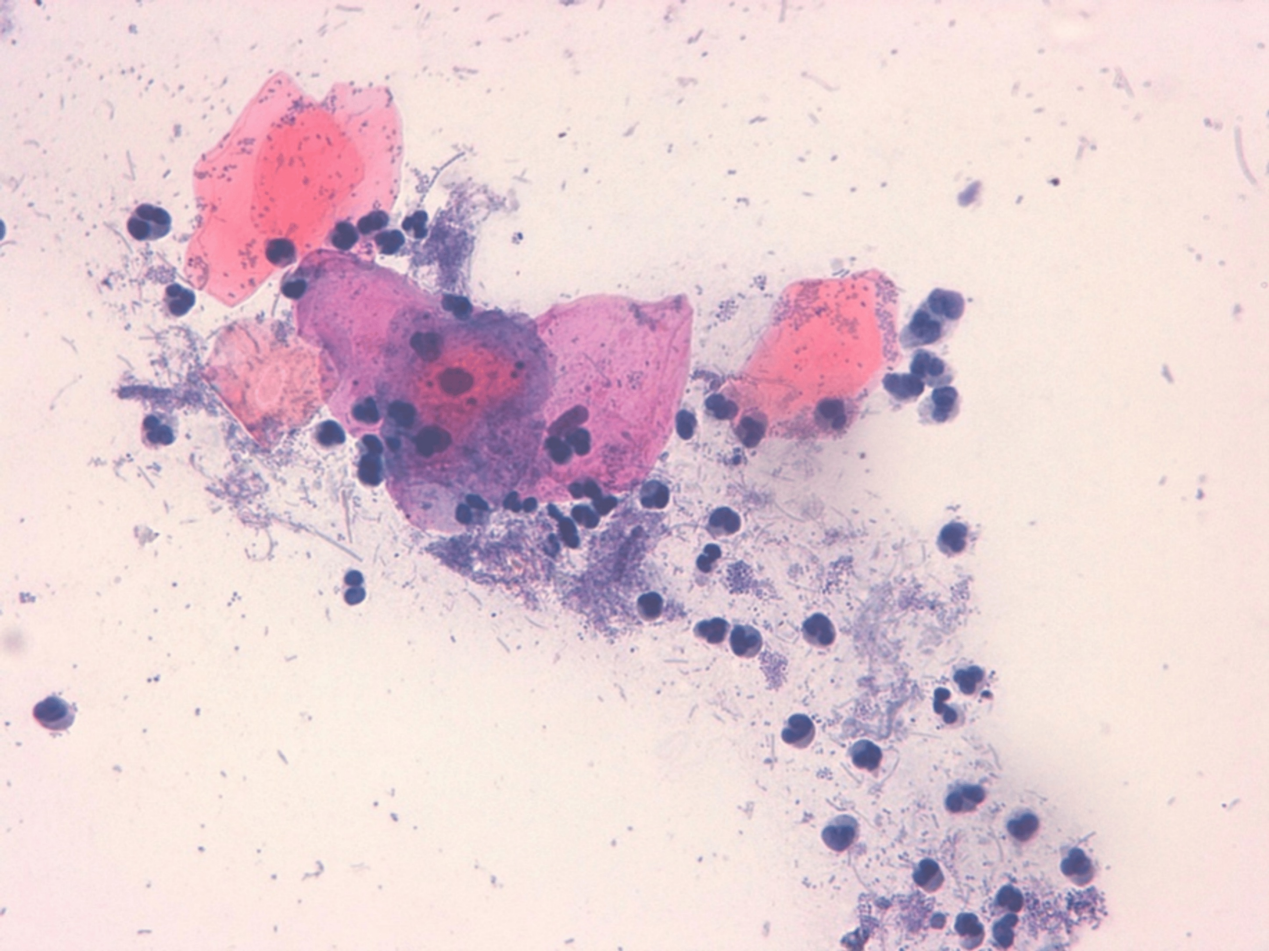
Microphotograph of a buccal smear from a cigarette smoker stained with the Papanicolaou technique (×40), demonstrating inflammatory cell infiltration.

**Figure 4:**
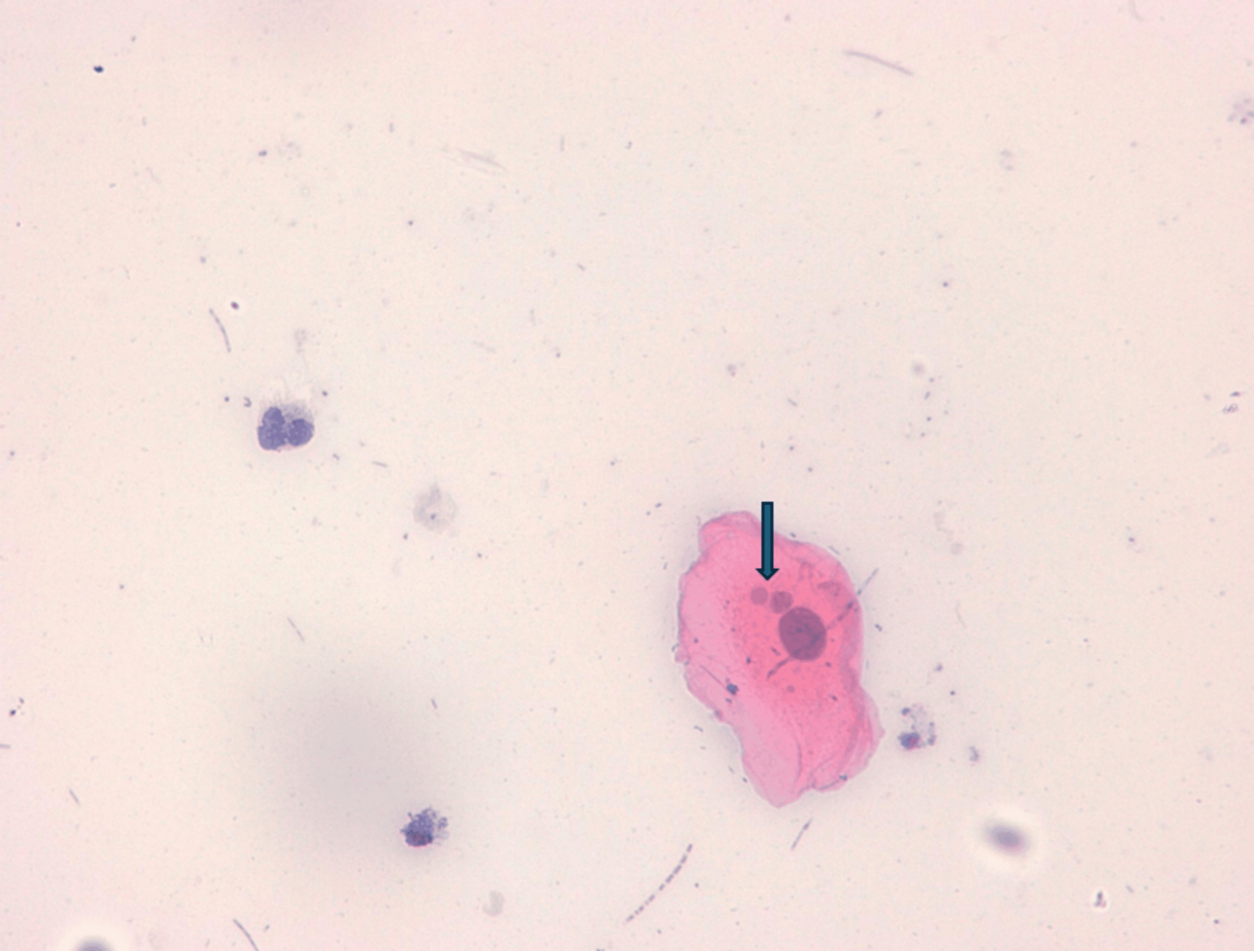
Microphotograph of a buccal smear from a cigarette smoker stained with the Papanicolaou technique (×40), demonstrating binucleated epithelial cell (arrow).

**Figure 5:**
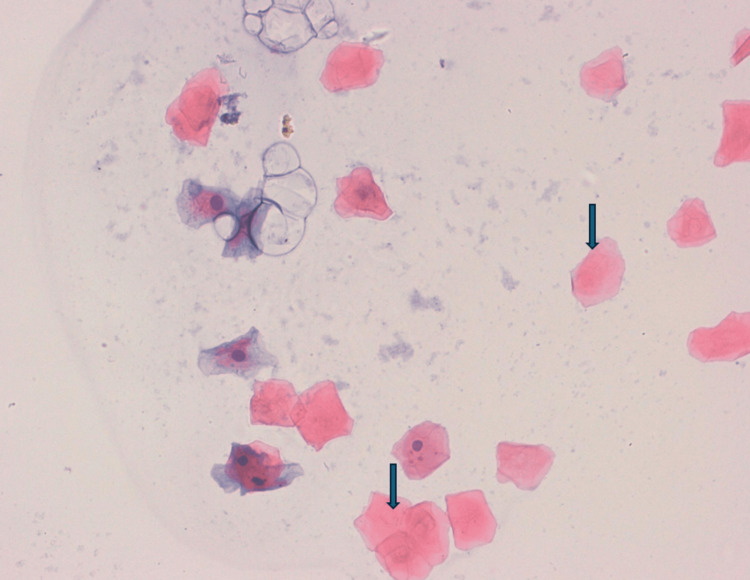
Microphotograph of a buccal mucosal smear stained using the Papanicolaou technique (×40), showing clusters of superficial squamous epithelial cells with dense eosinophilic cytoplasm, suggestive of hyperkeratinization.

Figures 2-5 illustrate some examples of smears stained using the Papanicolaou technique.

## Discussion

The findings of the present study demonstrate that both tobacco use (in its various forms) and frequent consumption of spicy foods are associated with significant cytological alterations in the oral mucosa, even in clinically normal tissues. The higher prevalence of abnormalities observed among smokers (48%) and spicy food consumers (34%), compared with the control group (20%) (p < 0.05), suggests that these lifestyle factors may be linked to subclinical cellular alterations detectable through exfoliative cytology.

A particularly notable finding was the higher prevalence of nuclear abnormalities among e-cigarette users, including cellular atypia (15%) and binucleation (8.8%). Although e-cigarettes are often considered a less harmful alternative to conventional smoking, the present findings suggest a higher prevalence of nuclear alterations in this subgroup. However, these findings should be interpreted with caution, given the relatively smaller subgroup size (n = 80) and the cross-sectional design of the study, which limits causal inference.

This observation is in line with recent studies suggesting that exposure to e-cigarette aerosols, which may contain substances such as formaldehyde and acrolein, may induce oxidative stress and cellular changes in oral epithelial cells [10]. Moreover, binucleation has been described as a cytological feature associated with altered cell division; however, its clinical significance in this context requires further investigation.

In contrast, spicy food consumers exhibited the highest prevalence of inflammatory changes (26%), supporting the role of dietary irritants in modulating oral epithelial responses. Capsaicin, the primary bioactive component in spicy foods, has been shown to activate TRPV1 receptors, which play a key role in mediating nociceptive and inflammatory responses [11]. Repeated exposure to capsaicin may contribute to epithelial irritation and low-grade inflammation in oral tissues [12]. The observed hyperkeratinization (12%) in this group may represent an adaptive epithelial response to repeated irritation, similar to changes reported in tobacco users [13].

Cigarette and shisha smokers also demonstrated elevated levels of inflammatory changes (23.3% and 22%, respectively), which may be attributed to the well-documented effects of tobacco smoke constituents. Tobacco smoke contains a wide range of carcinogenic compounds, including polycyclic aromatic hydrocarbons and nitrosamines, which are known to disrupt cellular homeostasis and promote inflammatory and cytological alterations [14]. Furthermore, the presence of cellular atypia among shisha users challenges the common misconception that water-pipe smoking is less harmful. Despite the filtration effect of water, prolonged exposure during shisha sessions may result in significant intake of carbon monoxide, heavy metals, and other toxic compounds [15].

The demographic profile observed in this study, particularly the higher proportion of smokers within the 18-30-year age group, reflects a growing trend of tobacco and alternative smoking product use among young adults [16]. This highlights the need for increased awareness and further investigation into the impact of these exposures on oral health.

Importantly, the use of Papanicolaou staining in this study proved to be a sensitive, non-invasive, and cost-effective method for detecting early cytological alterations. This supports its potential utility as a screening approach for identifying subclinical mucosal changes in individuals exposed to risk factors [17].

This study has several limitations. The cross-sectional design restricts the ability to establish causal relationships. The relatively smaller e-cigarette subgroup may limit the generalizability of subgroup findings. In addition, detailed quantitative exposure measures were not assessed separately for e-cigarette users. Potential confounding factors, including oral hygiene, alcohol consumption, and other lifestyle variables, were not fully controlled. Furthermore, samples were collected from multiple oral sites but analyzed collectively, precluding site-specific comparisons. Finally, the study relied on exfoliative cytology without histopathological confirmation.

Future longitudinal studies with larger sample sizes and more detailed exposure assessment are recommended to better understand the clinical significance of these findings.

## Conclusions

The present study demonstrates that tobacco use, including e-cigarette exposure, is associated with a higher prevalence of cytological alterations in the oral mucosa, while frequent consumption of spicy foods appears to be associated primarily with inflammatory changes. These findings should be interpreted with caution, given the cross-sectional design of the study and the limitations in exposure assessment. The observed cytological changes are suggestive of alterations that warrant further investigation rather than definitive indicators of dysplastic progression. Further longitudinal studies are needed to clarify the potential clinical significance of these findings.
